# From Plan to Practice: Challenges in Implementing Advance Care Planning in Care Home Residents

**DOI:** 10.7759/cureus.119376

**Published:** 2026-11-09

**Authors:** Sally Irwin, Sibonokuhle Moyo, Rebecca Walsh, Anna Steel

**Affiliations:** 1 Geriatrics, Barnet Hospital, Royal Free London NHS Foundation Trust, London, GBR; 2 Geriatrics, Royal Free London NHS Foundation Trust, London, GBR; 3 Haematology, St Bartholomew’s Hospital, Barts Health NHS Trust, London, GBR

**Keywords:** advance care planning, end-of-life-care, frailty syndrome, palliative care, palliative care and advance care planning (acp), treatment escalation plans

## Abstract

Advance care planning is a process where patients and their families can discuss and document their wishes and preferences for future care with healthcare professionals. However, the existence of an advance care plan (ACP) does not guarantee its implementation. We present the case of a 98-year-old woman living with severe frailty who developed a diabetic foot infection with dry gangrene, highlighting the challenges in implementing ACP and the gaps between documented care preferences and clinical practice. Despite an ACP to avoid hospital admission, the patient was transferred to hospital when her toes developed black discolouration. Shortly afterward, when she deteriorated in the care home, staff again considered hospital transfer, highlighting challenges in implementing ACPs within community settings. This case demonstrates the importance of clear escalation plans, effective communication between healthcare providers and care homes, and ongoing support for care home staff in implementing ACPs.

## Introduction

Advance care planning is a process where patients and their families can discuss and document their wishes and preferences for future care with healthcare professionals. Discussions may cover what matters most to the individual, their preferred place of care and death, and their preference regarding future medical treatment. For patients who lack mental capacity and do not have an advance care plan (ACP) in place, family members can play an important role in communicating their relative's previously expressed wishes, values and preferences, thereby informing healthcare professionals' best-interests decisions [1,2]. 

The National Institute for Health and Care Excellence (NICE) emphasises the importance of sharing ACP documentation with relevant people involved in a person’s care. This is particularly important during transitions between care settings, such as discharge from hospital to care home [1]. However, the existence of an ACP does not guarantee its implementation. Barriers include insufficient staff training, inadequate documentation and communication between healthcare settings, and limited time and resources [3]. 

We present the case of a 98-year-old woman living with severe frailty, who lacked mental capacity, with decision about her care made in her best interest. This case explores the challenges of implementing ACP, focusing on the gaps between documented care preferences and clinical practice. We discuss care home staff confidence in managing clinical deterioration, the role of family involvement in difficult decisions, and the importance of coordinated community palliative care in achieving a comfortable death in a care home. 

## Case presentation

A 98-year-old woman living with severe frailty, who was bedbound, non-verbal, with hearing and visual impairments, and lacked mental capacity, presented to the ED. She had a past medical history of asthma, hypertension, heart failure, osteoporosis, and type 2 diabetes mellitus treated with insulin. Two years prior, a percutaneous endoscopic gastrostomy (PEG) tube had been inserted for nutritional support due to poor oral intake and recurrent silent aspiration. 

One year prior to her final ED attendance, an ACP stating that she should not be readmitted to hospital had been discussed with her family and documented. Subsequently, the patient developed worsening erythema and pain in her right foot. Suspecting a foot infection, possibly cellulitis, her General Practitioner (GP) commenced oral antibiotics, administered via her PEG, in an attempt to manage her condition in the care home in keeping with her ACP. Despite treatment, her second and third right toes developed progressive black discolouration. Despite the existing ACP, care home staff felt unable to manage this confidently and transferred her to the ED.

At her final ED attendance, the patient was diagnosed with a diabetic foot infection with dry gangrene and started on intravenous (IV) antibiotics. She had a history of peripheral artery disease and had undergone stent insertion several years previously. Given her severe frailty, surgical intervention was not considered possible. Her ACP was discussed again with her family, and it was agreed that she should not be readmitted to hospital in the future. This plan was communicated to her GP and care home. She was discharged to her care home on IV antibiotics under the hospital-at-home team, with community podiatry input. 

When the patient later became symptomatic in the care home, staff again considered hospital readmission. Her daughter advocated for an alternative approach, requesting an assessment by the Rapid Response community team, which provides urgent assessment and support in patients' homes to help manage acute deterioration and avoid hospital admissions where appropriate. Despite being provided with the contact number, there was initial confusion between the care home and the Rapid Response team about this referral pathway. However, after some delay, the Rapid Response team came and assessed the patient. The patient was managed with anticipatory medication and subsequently died comfortably in the care home, with her family at her bedside. 

## Discussion

During the patient's final hospital attendance, an ACP was discussed with her family, documented and shared with the care home. The plan specified that the patient was not to be re-admitted to hospital and that her care should focus on maintaining comfort within the care home. Staff were advised to contact the Rapid Response team, a service able to assess and treat patients in the community and facilitate urgent clinical advice from the patient's GP and local Consultant Geriatrician. Despite this guidance, when the patient subsequently deteriorated, care home staff initially lacked confidence in managing her condition within the community and suggested hospital transfer. 

Similar challenges have been identified in the literature. Harrad-Hyde et al. reported that care home staff experienced uncertainty regarding their ability to meet residents' needs within the care home environment, concerns about making inappropriate decisions and potential repercussions, and difficulties interpreting ACPs that lacked specific guidance for different clinical situations [4,5]. A systematic review of end-of-life care in UK care homes similarly identified challenges relating to staff knowledge and confidence, alongside difficulties accessing specialist palliative care support within the community [6]. These findings may help explain the gap between the patient's documented ACP and the care home's initial response to her deterioration. 

Care home staff and other community healthcare professionals may experience difficulties implementing ACPs when documentation lacks clear guidance regarding the management of anticipated clinical complications and appropriate escalation pathways within the care home setting. In the absence of such guidance, hospital referral may be perceived as the safest or only available option, particularly when staff lack confidence in managing clinical deterioration in the community. Although ACPs cannot anticipate every potential complication, providing individualised management plans and clearly documented escalation pathways may help staff make informed decisions that align with patients' documented preferences and goals of care. 

This case highlights that documenting and sharing an ACP may be insufficient without the confidence, support and infrastructure required to implement it during episodes of clinical deterioration. Clear escalation pathways, staff education and timely access to clinical advice may help care homes manage residents safely within the community while respecting documented care preferences. 

A further barrier identified in the literature, although not evident in this case, is that family members may request hospital transfer despite a resident's documented preferences for care to remain within the care home. This can create additional challenges for staff attempting to implement ACPs [4,5]. 

It is important to recognise that an ACP focused on avoiding hospital readmission does not necessarily mean withholding active treatment. Rather, treatment may still be provided when clinically appropriate, with decisions guided by the patient's goals of care, the anticipated benefits and burdens of treatment, and overall comfort. In this case, discharge from hospital was facilitated by the community Hospital at Home service, enabling the patient to receive antibiotic treatment within her care home in accordance with the wishes documented in her ACP. Both the care home and the patient's daughter were provided with copies of the ACP. When the patient's condition subsequently deteriorated, her daughter was able to advocate for her mother's wishes and request alternative escalation options that did not require hospital admission. This demonstrates how treatment can be actively delivered in the community while remaining consistent with an individual's documented preferences. 

The successful avoidance of hospital readmission in this case is consistent with evidence suggesting that ACP interventions may reduce hospitalisations. Houben et al. reported that successful ACP interventions were associated with reduced hospital admissions [7]. Similarly, in a study examining the implementation of ACP within care homes, residents reported improvements in the quality of care they received. Residents were actively involved in ACP discussions alongside their relatives and were given opportunities to express their preferences regarding resuscitation, hospital readmission and other medical interventions [8]. This collaborative approach helped ensure that residents' wishes were recognised and incorporated into future care planning, suggesting that ACP can be implemented successfully within care home settings [8]. 

Capacity, best interests and family involvement are central to ACP, as it provides an opportunity to document a person's wishes while they have the capacity to express them. Capacity is both decision-specific and time-specific, and all adults are presumed to have capacity unless it is established otherwise [9]. When a person lacks capacity to make a particular decision, decisions should be made in their best interests, considering their past and present wishes, feelings, beliefs and values, while consulting those close to them [9]. The involvement of family members can therefore support ACP as a shared decision-making process; family members can provide information regarding a person's wishes and preferences for care and treatment, while responsibility for clinical decision-making remains with the healthcare team [10]. 

There is also an important emotional aspect to ACP that clinicians should recognise. Discussions surrounding ACP may be unfamiliar to people outside healthcare settings, and family members may experience considerable discomfort when engaging in these conversations [11]. In this case, the patient's next of kin had not previously heard of ACP before discussions with the Consultant Geriatrician, highlighting that many families may have limited awareness of the purpose and role of advance care planning. Reviews have identified several ways in which family factors can influence ACP discussions. Family members may avoid conversations about death and dying because they find them distressing, making ACP discussions more difficult to initiate. In addition, the emotional impact of a loved one's deterioration has been recognised as a barrier to effective engagement with ACP discussions [12]. 

## Conclusions

Advance care planning is a complex but valuable approach to facilitating treatment-escalation decisions and end-of-life care for patients living with severe frailty. ACP has been associated with reduced hospitalisations while also supporting treatment plans that align as closely as possible with patients' values and preferences. However, this case demonstrates that the successful implementation of an ACP depends on more than simply documenting a person's wishes. Care home staff may lack confidence in managing clinical deterioration outside hospital and may feel uncomfortable following recommendations against hospital readmission without clear support and escalation pathways. Improved communication between care homes, community services, families and healthcare professionals, alongside timely clinical support, clear documentation and staff training, may enhance ACP implementation in practice. This case demonstrates that successful ACP implementation depends not only on recording patients' wishes but also on ensuring that those responsible for their care are supported to act on those wishes when clinical deterioration occurs. 
